# Effects of soy milk consumption on gut microbiota, inflammatory markers, and disease severity in patients with ulcerative colitis: a study protocol for a randomized clinical trial

**DOI:** 10.1186/s13063-020-04523-8

**Published:** 2020-06-23

**Authors:** Omid Sadeghi, Alireza Milajerdi, Seyed Davar Siadat, Seyed Ali Keshavarz, Ali Reza Sima, Homayoon Vahedi, Peyman Adibi, Ahmad Esmaillzadeh

**Affiliations:** 1 Students Scientific Research Center, Tehran University of Medical Sciences, Tehran, Iran; 2 Department of Community Nutrition, School of Nutritional Sciences and Dietetics, Tehran University of Medical Sciences, P.O. Box 14155-6117, Tehran, Iran; 3 Department of Mycobacteriology and Pulmonary Research, Microbiology Research Center, Pasteur Institute of Iran, Tehran, Iran; 4 Department of Clinical Nutrition, School of Nutritional Sciences and Dietetics, Tehran University of Medical Sciences, Tehran, Iran; 5 Digestive Disease Research Institute, Shariati Hospital, Tehran University of Medical Sciences, Tehran, Iran; 6 Integrative Functional Gastroenterology Research Center, Isfahan University of Medical Sciences, Isfahan, Iran; 7 Obesity and Eating Habits Research Center, Endocrinology and Metabolism Molecular Cellular Sciences Institute, Tehran University of Medical Sciences, Tehran, Iran; 8 Food Security Research Center, Department of Community Nutrition, School of Nutrition and Food Science, Isfahan University of Medical Sciences, Isfahan, Iran

**Keywords:** Soy milk, Inflammation, Microbiota, RCT, Ulcerative colitis

## Abstract

**Background:**

Several strategies are recommended to alleviate clinical symptoms of ulcerative colitis (UC). Soy milk may affect UC through its anti-inflammatory properties. However, no study has examined the effects of soy milk consumption on gut microbiota and inflammatory biomarkers in patients with UC. The current study will be done to examine the effects of soy milk consumption on UC symptoms, inflammation, and gut microbiota in patients with UC.

**Methods:**

This study is a randomized clinical trial, in which thirty patients with mild to moderate severity of UC will be randomly allocated to receive either 250 mL/day soy milk plus routine treatments (*n* = 15) or only routine treatments (*n* = 15) for 4 weeks. Assessment of anthropometric measures and biochemical indicators including serum concentrations of high-sensitivity C-reactive protein (hs-CRP), tumor necrosis factor-α (TNF-α), interleukin-1β (IL-1β), and interferon gamma (IFN-γ) will be done at the study baseline and end of trial. In addition, the quantity of butyrate-producing bacteria including *Clostridium* cluster IV, *Faecalibacterium prausnitzii*, and *Roseburia* spp.; prebiotic bacteria including *Lactobacillus* spp. and *Bifidobacteria* spp.; and mucus-degrading bacteria including *Akkermansia muciniphila*, *Bacteroides fragilis*, and *Ruminococcus* spp., as well as calprotectin and lactoferrin levels, will be explored in fecal samples. Also, the *Firmicutes* to *Bacteroidetes* ratio which is of significant relevance in human gut microbiota composition will be assessed.

**Discussion:**

Altered gut microbiota has been reported as an important contributing factor to inflammation in patients with inflammatory bowel disease (IBD). Soy milk contains several components such as phytoestrogens with potential anti-inflammatory properties. This product might affect gut microbiota through its protein and fiber content. Therefore, soy milk might beneficially affect systemic inflammation, gut microbiota, and then clinical symptoms in patients with UC.

**Trial registration:**

Iranian Registry of Clinical Trials (www.irct.ir) IRCT20181205041859N1. Registered on 27 January 2019.

## Background

Inflammatory bowel disease (IBD) is a chronic gastrointestinal disease associated with high disability and a high cost for patients as well as for the health care system [1–4]. Epidemiological studies have shown that more than 10 million people suffer from IBD worldwide [4]. National estimates in Iran showed that 40 persons among 100,000 people are affected [5]. The most common type of IBD is ulcerative colitis (UC) that is characterized by inflammation and ulceration of the mucosal layer of the colon, and its patients usually suffer from rectal bleeding (with or without mucus), diarrhea, tenesmus, and abdominal pain when the disease relapsed [6, 7].

Several studies have been done to find an appropriate strategy to control the disease symptoms and inflammation in patients with UC [8]. Patients with UC might take benefit from the consumption of fish oils, polyunsaturated fatty acids, and probiotics [9–11]. Soy and its products have been shown to have anti-inflammatory properties, particularly in patients with diabetes mellitus and cardiovascular diseases [12–15]. Due to the inflammatory nature of UC, consumption of soy products may affect UC disease symptoms as well. However, to our knowledge, no study was done to assess the efficacy of soy consumption on inflammatory markers and clinical outcomes in UC patients. In an experimental study, consumption of soy milk, fermented with six lactic acid bacteria strains, prevented the shortening of colon length, breaking of epithelial cells, lowering liver and thymus weights, and enlargement of spleen in mice fed dextran sodium sulfate (DSS) used for inducing IBD [16]. In another study on DSS-treated mice, soy protein administration reduced weight loss, colon shortening, splenomegaly, and colonic inflammation [17].

A large number of studies have shown a link between gut microbiota and UC [18–20]. In a review article, Bellavia et al. concluded that imbalanced gut microbiota is involved in UC pathogenesis [18]. Moreover, abnormalities of intestinal microbiota in UC patients, compared with healthy individuals, were reported in a case-control study [19]. These abnormalities may induce gut inflammation and consequently systemic inflammation [20]. In addition, the link between gut microbiota abnormalities and UC symptoms has been shown in earlier studies [21–23]. Balancing the gut microbiota through fecal microbiota transplantation can relief UC symptoms [23]. There are several investigations that indicated favorable association of soy products with balanced gut microbiota; of them, a cross-sectional study revealed that lower intake of soy drinks was associated with a reduced abundance of potentially beneficial bacteria and increased potentially harmful bacteria in the colonic mucosa of healthy individuals [24]. In a randomized clinical trial, Fernandez-Raudales et al. reported that soy milk consumption resulted in a potentially beneficial alteration in the *Firmicutes* to *Bacteroidetes* ratio in the gut [25]. Soy protein administration, compared with cow milk protein intake, resulted in a beneficial change in gut microbiota in an experimental study as well [26].

Among all soy products, soy milk can be a good choice for UC patients because they are recommended to limit their consumption of milk [27], which might prohibit them meeting their calcium requirement. Although not a rich source of calcium, soy milk can provide at least part of calcium requirements in these patients [16]. Therefore, we designed this study to examine if soy milk consumption can influence gut microbiota, inflammatory markers, quality of life, symptoms, and disease severity in patients with UC.

## Material and methods

### Participants

This is a randomized clinical trial which will be done in Tehran, Iran, in 2018–2019. Outpatients with UC will be recruited from the gastrointestinal (GI) clinic of the Shariati Hospital, Tehran, Iran. UC will be confirmed by a gastroenterologist through the use of colonoscopy and laboratory findings [28].

### Inclusion criteria

We will include (1) patients with mild to moderate UC, (2) those who are between 20 and 60 years, (3) patients at the remission stage of disease with stable medication therapy, and (4) those with body mass index (BMI) of 18.5–30 kg/m^2^. To determine disease severity, the partial-Mayo scale will be applied. Based on this scale, scores of 3–8 are considered as mild to moderate UC [29].

### Non-inclusion criteria

Patients will not be included if they (1) changed type or dosage of their medicines over the last 3 months; (2) were hospitalized in the last 3 months; (3) were diabetics or were affected by celiac or other gastrointestinal diseases including cancers and infectious diseases; (4) were pregnant or lactating; (5) consumed antibiotics, pre- or probiotic products, multi-vitamin, and mineral supplements during the last 3 months; and (6) were current smokers.

### Exclusion criteria

**Fig. 1:**
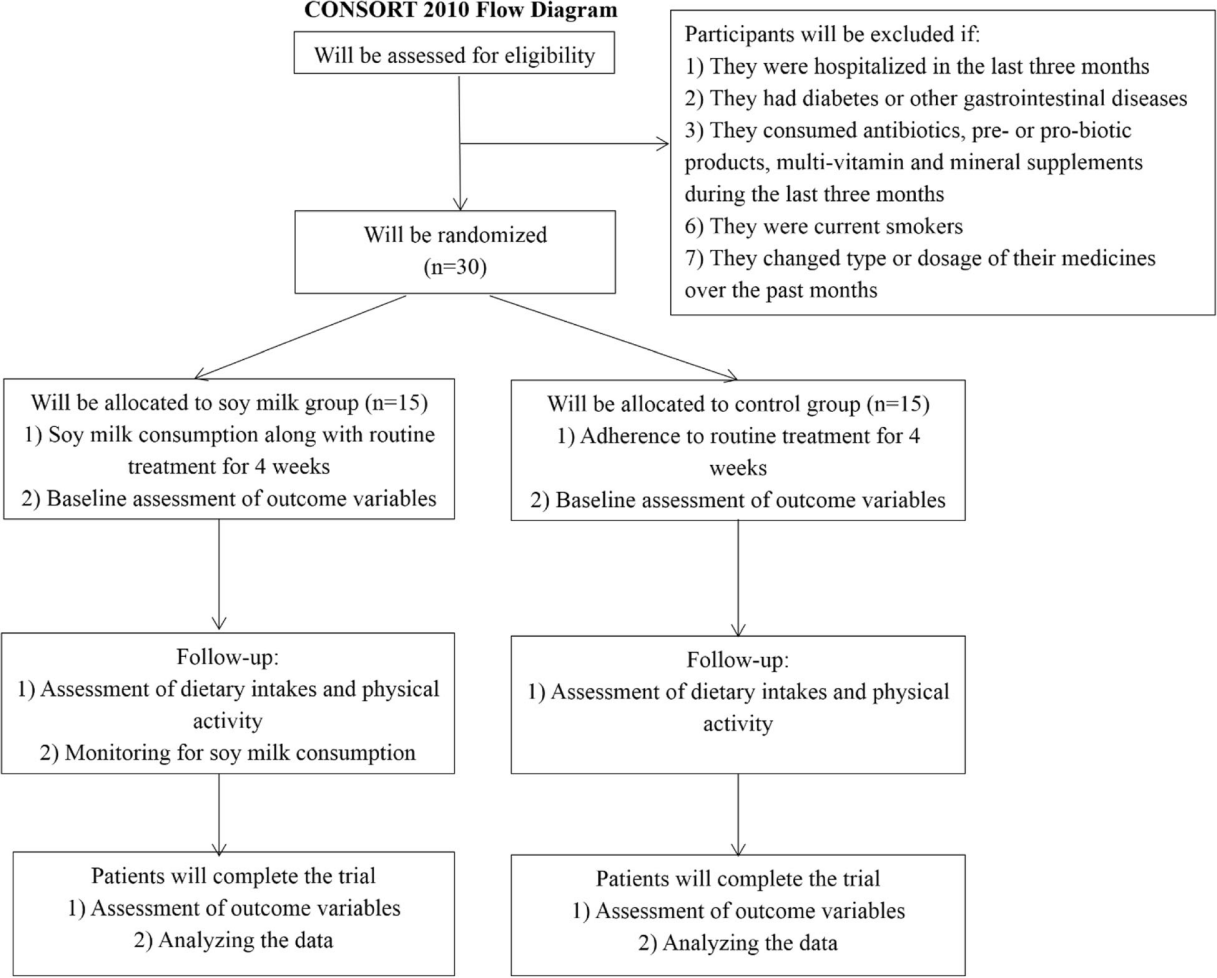
Study flowchart

We will exclude individuals who change the type or dosage of their medicines during the intervention, those that are not willing to continue intervention, and individuals who suffer from probable complications related to soy milk consumption. Figure 1 shows the study flowchart.

All participants will read the terms and conditions written in an informed consent (Supplementary file, section A), and optionally, they can accept to participate in the current trial. The ethics committee of Tehran University of Medical Sciences approved the study. Moreover, this clinical trial was registered in the Iranian Registry of Clinical Trials (www.irct.ir) on 27 January 2019 with code number of IRCT20181205041859N1.

### Sample size calculation

Considering the type I error of 5% (*α* = 0.05) and type II error of 20% (*β* = 0.20, power = 80%) and fecal concentrations of calprotectin as the key variable, we manually, without the use of any software, calculated required sample size using the following formula [30]:

$$ n=\frac{2\left[{\left(a+b\right)}^2\times {\upsigma}^2\right]}{{\left({\mu}_1-{\mu}_2\right)}^2} $$

*n* = sample size in each group.

*μ*_1_ = mean for fecal calprotectin in the intervention group (we considered it as 424 μg/g based on the study of Morshedzadeh et al. [31]).

*μ*_2_ = mean for fecal calprotectin in the control group (which was considered as 602 μg/g based on the study of Morshedzadeh et al. [31]).

σ = variance (SD) for mean concentrations of calprotectin, which was considered as 141 (the average SDs reported for fecal calprotectin in the control and intervention groups of Morshedzadeh et al.’s study [31]).

*a* = conventional multiplier for alpha = 0.05 that was 1.96.

*b* = conventional multiplier for power = 0.80 that was 0.842.

Overall, based on the formula and given a 50% drop-out in each group, we will need a sample size of 15 persons for each group. Due to frequent visits (about 6 visits) that each participant would have throughout the study, we considered the drop-out rate as much as 50%. However, we would try our best to keep the drop-outs as low as possible.

### Study design and intervention

**Fig. 2:**
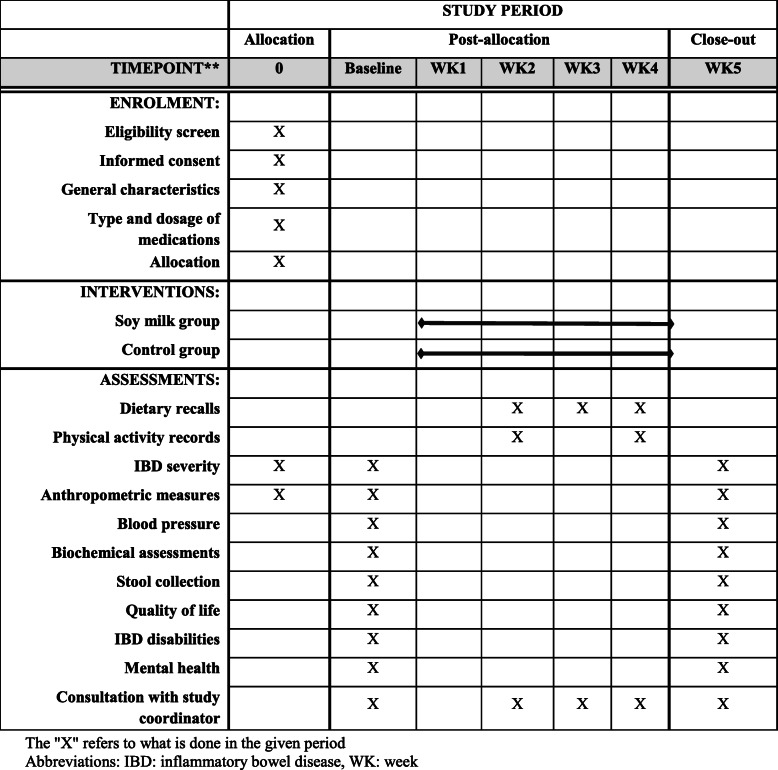
Standard Protocol Items: Recommendations for Interventional Trials (SPIRIT) chart of the enrolments and assessments during randomized controlled trials. The “X” refers to what is done in the given period. Abbreviations: IBD, inflammatory bowel disease; WK, week

The diagram for Standard Protocol Items: Recommendations for Interventional Trials (SPIRIT) is shown in Fig. 2. After recruiting participants, participants will be stratified based on age (20 to 40 and 40 to 60 years), gender (male/female), BMI (18.5 to 24.9 and 25 to 30), and type of medicines (anti-inflammatory and immunosuppressant) into different blocks and will be randomly allocated to the intervention or control groups. For each patient in a certain block, a matched person in terms of abovementioned variables would be placed in that block. Then, these two patients in a given block would be randomly assigned to the intervention and control groups. To do randomization, an identification code will be given to each eligible patient, and then, code of patients with the same age, gender, BMI, and medication will be stated in the lottery container, and finally, patients with the same conditions will be randomly assigned to the intervention or control groups. Random allocation will be done by a person who is unaware about the aim of our study. Patients in the intervention group (*n* = 15) will receive 250 mL/day preservative-free soy milk along with routine treatments that they were taking for their condition. Participants in the control group (*n* = 15) will receive only routine treatments. The intervention will last for 4 weeks. The assessment of primary and secondary outcome variables will be done at the study baseline and end of trial. Participants in both groups will also receive usual dietary recommendations for IBD patients based on European Society for Parenteral and Enteral Nutrition (ESPEN) guideline [32]. This guideline will be explained to patients by an experienced nutritionist. Given that preservative-free soy milk will be expired within 12 days after production, three 1-L bottles of soy milk will be delivered to patients in intervention group every 12 days (at the study baseline, day 12, and day 24). At the study baseline and day 12, patients in the intervention group will received 3 1-L bottles of soy milk (used during 12 days), and at day 24, they will received 2 1-L bottles of soy milk (used during 8 days). Due to the unfavorable taste of soy milk, patients in intervention group will be asked to consume it at night before sleep. They should consume a 250-mL glass of soy milk every night. Participants will be instructed in a class, prior to the start of trial, about the quantity of soy milk they have to consume in a day. A standard glass (which contained 250 mL soy milk) will be shown to participants, and all will be asked to use the same glass to make sure that the daily portion they are consuming is the one the investigators requested. To control this, participants will be contacted every day and they will be asked to send us the photo of the glass they are using for soy milk drinking. At each delivery of soy milk bottles, patients will be monitored for soy milk consumption and will be asked to give back the empty bottles. Participants’ compliance will be assessed using the following formula: (number of used packages/all given packages) × 100. In addition to assessment of compliance, consumption of soy milk will be monitored by phone call every week. In addition, collecting three 1-day dietary recalls from participants throughout the study can further help computing the compliance to soy milk intake. Participants will be asked not to change their lifestyle, dietary habits, and medicines during the study.

To assess dietary intakes throughout the study, we will fill 3 1-day dietary recalls (including two working days and a non-working day) at weeks 2, 3, and 4 of intervention by telephone interview. Dietary recall format is presented in Supplementary file, section B. To complete the dietary recalls, participants will be asked to report dietary intakes based on household measures. Then, we will convert household measures to grams using available booklets. The average of dietary intakes in these 3 days will be considered as participants’ dietary intakes throughout the intervention. To obtain nutrient intakes on the basis of these food recalls, we will use Nutritionist IV software (based on US National Nutrient Databank) modified for Iranian foods.

In addition, 2 1-day physical activity records (including a working day and a non-working day) will be gathered from all patients at weeks 2 and 4 of intervention to assess physical activity during the study. The format of physical activity record is shown in Supplementary file, section C. All participants will be educated how to record their activities throughout the study before the start of the trial. Furthermore, patients in both groups will be asked not to change their physical activity throughout the trial.

### Assessment of variables

A standard questionnaire will be used to obtain information on age, gender, education, economic status, medical history, medication use, smoking, and duration of UC. In addition, primary outcomes including serum concentrations of inflammatory biomarkers, fecal concentrations of calprotectin and lactoferrin, quantity of selected bacteria, IBD severity, and disability as well as secondary outcomes including complete blood counts (CBC), erythrocyte sedimentation rate (ESR), quality of life, mental health, anthropometric measures, and blood pressure will be measured at the beginning and end of study.

### Biochemical assessments

After 12 h of fasting, a 10-mL venous blood sample will be taken from each patient at the study baseline and end of trial. Total CBC, hemoglobin concentrations, and hematocrit will be measured by a cell counter. Erythrocyte sedimentation rate will be measured using the Wintergreen method. Then, serum will be isolated from the whole blood and will be stored at − 70 °C until further analysis. Serum concentrations of inflammatory cytokines, including high-sensitivity C-reactive protein (hs-CRP), Tumor necrosis factor-α (TNF-α), interleukin-1β (IL-1β), and interferon gamma (IFN-γ) will be measured by the enzyme-linked immunosorbent assay (ELISA) commercial kits. Earlier studies have shown that hs-CRP, TNF-α, IL-1β, and IFN-γ concentrations are positively associated with severity of IBD, particularly UC [33].

### Gut microbiota and fecal calprotectin

From each individual, 10-g stool sample will be taken to examine gut microbiota and fecal levels of calprotectin and lactoferrin at the study baseline and end of trail. A clean and screw-top container labeled with patient’s code and the date will be given to each patient for fecal sample collection. Participants will be asked to do fecal sampling in the morning. To decrease the changes in the quantity of bacteria in fecal samples after sampling, samples will be frozen at − 70 °C within 2 h after sampling. To take care of this, we will use three approaches: First, we will ask patients to come to the study lab at the day of stool sampling. All the necessary facilities will be available to do fecal sampling in the lab. Second, if the first approach did not work for a patient, we will ask him/her to bring the sample within2 h to the lab. Third, if he/she was not able to do the second approach, we will ask patients to call the study investigators and then somebody from the team would go the patients’ home and take the sample to the lab. The samples will be kept at − 70 °C until further analysis. Total DNA from stool samples will be extracted using the QIAamp (QIAGEN) commercial fecal DNA kits. In addition, the quantity of butyrate-producing bacteria including *Clostridium* cluster IV, *Faecalibacterium prausnitzii*, and *Roseburia* spp.; prebiotic bacteria including *Lactobacillus* spp. and *Bifidobacteria* spp.; and mucus-degrading bacteria including *Akkermansia muciniphila*, *Bacteroides fragilis, Ruminococcus* spp., as well as *Firmicutes* and *Bacteroidetes*, will be determined using the quantitative real-time polymerase chain reaction (qRT-PCR) which will be done by the LightCycler® 96 SW 1.1 instrument (Roche, Germany). Each reaction mixture contains SYBR Premix Ex Taq II (Takara, China), specific primers, and DNA template. In order to design primers, we will use sequence of primers used in the earlier studies [34, 35]. Selection of taxa was based on their correlation with UC and their effectiveness on gut microbiota balance [36]. It has been proposed that mucus-degrading bacteria have an important role in the etiology of UC which is defined as an intestinal mucosal disorder [36]. Also, most selected bacteria are involved in production of short-chain fatty acids (SCFAs), particularly butyrate, which are important metabolites in maintaining intestinal homeostasis [37, 38]. SCFAs are the important fuel for intestinal epithelial cells, are known to strengthen the gut barrier function, and activate signaling cascades that control immune functions and consequently intestinal inflammation [38]. Based on the previous studies, UC patients not only show reduced levels of SCFAs-producing bacteria in intestinal mucosa and feces, but the actual steady-state levels of SCFAs herein also appear to be less than healthy controls [39, 40]. Furthermore, in most clinical trials on patients with intestinal disorders such as UC or irritable bowel syndrome, these taxa were evaluated [34, 41].

In addition to fecal microbiota, fecal concentrations of calprotectin and lactoferrin will be measured using the ELISA commercial kits. Calprotectin is a calcium binding protein commonly derived from neutrophils and monocytes and in a less extent from macrophages [42]. Calprotectin is found in stool and plasma, and its concentration increases throughout inflammatory conditions, including IBD and infection [43]. Lactoferrin is a glycoprotein expressed by activated neutrophils [44]. This factor is sensitive and specific for detecting inflammation in chronic IBD [44].

### Anthropometric measures

Weight will be measured using a digital scale at the state of minimum clothing without shoes to the nearest 100 g. Standing height will be measured using a standard stadiometer, without shoes, to the nearest 0.5 cm. BMI will be determined as weight in kilograms divided by height in meters squared.

### Blood pressure

Systolic and diastolic blood pressures will be measured twice with a 15-min interval at the right arm using a mercury barometer calibrated by the Institute of Standardization and Industrial Research. Before measurement, patients will be asked to rest for 5 min. The average of two measurements will be considered as participants’ systolic and diastolic blood pressures.

### IBD severity

The severity of IBD will be examined using the 9-point partial Mayo score designed by Sutherland et al. (the questionnaire is available in Supplementary file, section D) [29]. This scale composes of three categories including rectal bleeding (0 = none, 1 = visible blood with stool less than half the time, 2 = visible blood with stool half of the time or more, 3 = passing blood alone), stool frequency (0 = normal, 1 = 1–2 stools/day more than normal, 2 = 3–4 stools/day more than normal, 3 = > 4 stools/day more than normal), and physician assessment (0 = normal, 1 = mild, 2 = moderate, 3 = severe) that each gets 0–3 score. By summing up these scores, we will have a total score of 0–9. Greater scores show higher severity of disease.

### Quality of life

We will use inflammatory bowel disease questionnaire-9 (IBDQ-9) to assess patients’ quality of life (the questionnaire is available in Supplementary file, section E) [45]. This questionnaire comprises 9 items that assess 4 dimensions including bowel and systemic symptoms, emotional status, and social functioning. Each item is scored from 1 to 7. Total score of this questionnaire ranges from 9 to 63; in which greater scores show better quality of life. Prior studies have shown that the questionnaire presents valid and reliable data on quality of life of IBD patients.

### IBD disabilities

To assess IBD-related disabilities, we will apply inflammatory bowel disease disability index (IBD-DI) that contains 28 questions on patients’ health, body functions, body structures and activities, participation, and environmental factors (the questionnaire is available in Supplementary file, section F) [46]. Details on scoring of IBD-DI were elaborated by Leong et al. [47]. The response of each item on the questionnaire is either dichotomous “yes” versus “no” or ordinal on a 1 to 5 Likert scale (1 being no difficulty and 5 being extreme difficulty). Scores from each question are combined to obtain total score of each domain and a final composite score of disability ranging from − 80 (maximum degree of disability) to 22 (no disability) with zero as the anticipated point of neutrality.

### Mental health

Based on previous publications, psychological disorders including depression, anxiety, and psychological distress are prevalent among IBD patients [48, 49]. Depression and anxiety will be screened using the Iranian validated version of Hospital Anxiety and Depression Scale (HADS) which is a brief and useful questionnaire to assess psychological disorders and to measure the symptoms and severity of anxiety disorders and depression (the questionnaire is available in Supplementary file, section G) [50]. It contains fourteen items and consists of two subscales: anxiety and depression. Each item includes a 4-point scale; higher scores indicate an increased level of anxiety and depression. Maximum score is 21 for anxiety and depression. To assess psychological distress, we will use the Iranian validated version of General Health Questionnaire (GHQ) that includes 12-items (the questionnaire is available in Supplementary file, section H) [51]. Each item provides a 4-point rating scale (less than usual, no more than usual, rather more than usual, or much more than usual). To calculate the total score of psychological distress for each participant, we will use the bimodal scoring method (0-0-1-1) [52, 53]. This method gives the total score ranging from 0 to 12; higher scores indicate a greater degree of psychological distress. Overall, our previous investigations revealed that the questionnaires provide relatively valid measures of mental health.

### Adverse events

Patients will be asked to report any adverse events that may have occurred throughout the study. Investigators will record these events.

### Statistical analysis

The Kolmogorov-Smirnov test will be used to examine normal distribution of variables. Log transformation will be conducted for the non-normally distributed data. To examine differences in qualitative variables between soy milk and control groups, we will use the chi-square test. We will apply the independent sample *t* test to detect differences in quantitative variables between the soy milk and control groups. In addition, mentioned test will be used to compare between-group changes in outcome variables. To do within-group comparison, we will use the paired-sample *t* test. Multivariate analysis of covariance (ANCOVA), as a general linear model, will be used to examine the effects of soy milk consumption on outcome variables. In this analysis, baseline value of outcome variables and potential confounding variables which are different between the intervention and control groups will be adjusted to avoid potential risk of bias and detect independent results. All statistical analyses will be done using the SPSS software version 18 (SPSS, Inc. Chicago, IL, USA). *P* < 0.05 will be considered significant.

## Discussion

Currently, some medications including amino salicylates, glucocorticosteroids, immune modulators, antibiotics, and anti-TNF drugs are prescribed for IBD patients, which cause severe adverse effects such as increasing anti-antibody reactions, risk of allergy, infection, and mutagenesis [54–56]. Thus, finding a food with therapeutic effects and without toxicity is of great importance for IBD patients. Soy products such as soy milk with anti-inflammatory properties are a good choice for inflammatory diseases including diabetes mellitus and cardiovascular diseases [12, 13, 57]. However, so far, no study has examined the effects of soy milk consumption on inflammatory markers and disease symptoms in patients with IBD. In addition, the beneficial effects of soy milk consumption on gut microbiota have been reported [58]; however, these effects have not been yet assessed in IBD patients. As recently stated, change in the gut microbiota has been a contributing factor to IBD incidence, particularly UC [59–61]. In addition, it has been shown that gut microbiota imbalance can affect disease symptoms [62].

In this study, we hypothesize that soy milk consumption might have a beneficial effect in IBD patients through increasing abundance of beneficial/commensal bacteria and reduction of mucus-degrading taxa. Soy milk contains 0.6 g/100 g fiber which can be considered as a prebiotic [63]. It has long been shown that prebiotics can favorably affect gut microbiota [64]. In an experimental study, soy milk administration resulted in an increase in *Firmicutes* to *Bacteroidetes* ratio in gut microbiota community of rats [58]. Also, soy milk intake had an increasing effect on *Lactobacillus* spp. which potentially protects against gut inflammation [65]. In addition to fiber, protein content of soy milk can affect gut microbial community [66, 67]. In an experimental study on golden Syrian hamsters, soy protein, compared with milk protein, could alter diversity of gut microbial community [68].

Patients with IBD cannot easily tolerate lactose-containing foods such as dairy products [68, 69]. Soy milk as a lactose-free milk might be a good alternative for dairy products in IBD patients. Overall, finding dietary strategies to improve disease severity and symptoms in IBD patients is of high importance. This approach could be much more cost-effective than prescription of medications for long-term use in these patients.

### Strengths

This is the first clinical trial investigating the effects of soy milk consumption on gut microbiota and inflammatory markers in patients with UC. It must be kept in mind that the intervention is of low cost and can easily be carried out in clinical practice. In this study, patients will be block matched with each other in terms of several variables that might influence the eventual findings. Several outcomes including subjective- and objective-based symptoms along with biochemical indicators will be examined in patients at the study baseline and end of trial. Moreover, compliance to the intervention will be assessed throughout the study.

We hypothesize that soy milk consumption can result in decreased inflammation through inducing changes in gut microbial community. Improvement in gut microbiota and inflammatory markers would lead to beneficial effects on disease symptoms and severity as well as quality of life.

### Limitations

Despite using validated questionnaires for assessment of dietary intakes, disease severity, disabilities, and quality of life, misclassification of participants cannot be fully excluded. Furthermore, change in gut microbiota during the sample collection phase is the most important concern for investigators. To avoid this, we will collect fecal samples in the laboratory and keep it immediately at − 70 °C. In addition, we would try our best to have all the fecal samples in the lab within the 2 h after sampling; however, some samples might arrive within > 2 h. One might argue that the sample size would be inadequate for this study; however, it must be kept in mind that several highly cost variables including six inflammatory biomarkers and 9 genes for quantitative real-time polymerase chain reaction (qRT-PCR) are supposed to be quantified in this study. Due to limited funding in developing countries, performing the study with a sample size greater than the one computed for this study would not be affordable for researchers. In addition, most studies on microbiota or fecal calprotectin have been done with a similar sample size [70–73].

## Supplementary information

**Additional file 1.**

## Data Availability

Not applicable

## Abbreviations

IBD: Inflammatory bowel disease
UC: Ulcerative colitis
BMI: Body mass index
CBC: Complete blood count
HADS: Hospital anxiety and depression scale
hs-CRP: Highly sensitive-C reactive protein
TNF-α: Tumor necrosis factor-α
IL-1β: Interleukin-1β
IFN-γ: Interferon gamma

## References

[1] Burisch J, Jess T, Martinato M, Lakatos PL (2013). ECCO-EpiCom. The burden of inflammatory bowel disease in Europe. J Crohns Colitis.

[2] Allen PB, Gower-Rousseau C, Danese S, Peyrin-Biroulet L (2017). Preventing disability in inflammatory bowel disease. Ther Adv Gastroenterol.

[3] Everhart JE, Ruhl CE (2009). Burden of digestive diseases in the United States part III: liver, biliary tract, and pancreas. Gastroenterology.

[4] Global, regional, and national incidence, prevalence, and years lived with disability for 301 acute and chronic diseases and injuries in 188 countries, 1990–2013: a systematic analysis for the Global Burden of Disease Study 2013. Lancet. 2015;386(9995):743–800. 10.1016/s0140-6736(15)60692-4. 10.1016/S0140-6736(15)60692-4 PMC4561509 26063472

[5] Malekzadeh MM, Vahedi H, Gohari K, Mehdipour P, Sepanlou SG, Ebrahimi Daryani N, Zali MR, Mansour-Ghanaei F, Safaripour A, Aghazadeh R (2016). Emerging epidemic of inflammatory bowel disease in a middle income country: a nation-wide study from Iran. Arch Iran Med.

[6] Abraham C, Cho JH (2009). Inflammatory bowel disease. N Engl J Med.

[7] Kamali M, Tavakoli H, Khodadoost M, Daghaghzadeh H, Kamalinejad M, Gachkar L, Mansourian M, Adibi P (2015). Efficacy of the Punica granatum peels aqueous extract for symptom management in ulcerative colitis patients. A randomized, placebo-controlled, clinical trial. Complement Ther Clin Pract.

[8] Shivashankar R, Lewis JD (2017). The role of diet in inflammatory bowel disease. Curr Gastroenterol Rep.

[9] Tatar EL, Das KM (2007). Improvement in ulcerative colitis symptoms after use of fish oil enemas. Gastroenterol Hepatol (N Y).

[10] Abraham BP, Quigley EMM (2018). A probiotic for ulcerative colitis: the culture wars continue. Dig Dis Sci.

[11] Sheikhi A, Shakerian M, Giti H, Baghaeifar M, Jafarzadeh A, Ghaed V, Heibor MR, Baharifar N, Dadafarin Z, Bashirpour G (2016). Probiotic yogurt culture Bifidobacterium animalis subsp. lactis BB-12 and Lactobacillus acidophilus LA-5 modulate the cytokine secretion by peripheral blood mononuclear cells from patients with ulcerative colitis. Drug Res (Stuttg).

[12] Miraghajani MS, Esmaillzadeh A, Najafabadi MM, Mirlohi M, Azadbakht L (2012). Soy milk consumption, inflammation, coagulation, and oxidative stress among type 2 diabetic patients with nephropathy. Diabetes Care.

[13] Rebholz CM, Reynolds K, Wofford MR, Chen J, Kelly TN, Mei H, Whelton PK, He J (2013). Effect of soybean protein on novel cardiovascular disease risk factors: a randomized controlled trial. Eur J Clin Nutr.

[14] Jheng HF, Hirotsuka M, Goto T, Shibata M, Matsumura Y, Kawada T. Dietary low-fat soy milk powder retards diabetic nephropathy progression via inhibition of renal fibrosis and renal inflammation. Mol Nutr Food Res 2017;61(3). doi: 10.1002/mnfr.201600461. 10.1002/mnfr.201600461 27748993

[15] Feizollahzadeh S, Ghiasvand R, Rezaei A, Khanahmad H, Hariri M (2017). Effect of probiotic soy milk on serum levels of adiponectin, inflammatory mediators, lipid profile, and fasting blood glucose among patients with type ii diabetes mellitus. Probiotics Antimicrob Proteins.

[16] Kawahara M, Nemoto M, Nakata T, Kondo S, Takahashi H, Kimura B, Kuda T (2015). Anti-inflammatory properties of fermented soy milk with Lactococcus lactis subsp. lactis S-SU2 in murine macrophage RAW264.7 cells and DSS-induced IBD model mice. Int Immunopharmacol.

[17] Bitzer ZT, Wopperer AL, Chrisfield BJ, Tao L, Cooper TK, Vanamala J, Elias RJ, Hayes JE, Lambert JD (2017). Soy protein concentrate mitigates markers of colonic inflammation and loss of gut barrier function in vitro and in vivo. J Nutr Biochem.

[18] Bellavia M, Tomasello G, Romeo M, Damiani P, Monte AI, Lozio L, Campanella C, Gammazza AM, Rappa F, Zummo G, Cocchi M (2013). Gut microbiota imbalance and chaperoning system malfunction are central to ulcerative colitis pathogenesis and can be counteracted with specifically designed probiotics: a working hypothesis. Med Microbiol Immunol.

[19] Noor SO, Ridgway K, Scovell L, Kemsley EK, Lund EK, Jamieson C, Johnson IT, Narbad A (2010). Ulcerative colitis and irritable bowel patients exhibit distinct abnormalities of the gut microbiota. BMC Gastroenterol.

[20] Saad MJ, Santos A, Prada PO (2016). Linking gut microbiota and inflammation to obesity and insulin resistance. Physiology (Bethesda).

[21] Vester-Andersen MK, Mirsepasi-Lauridsen HC, Prosberg MV, Mortensen CO, Träger C, Skovsen K, Thorkilgaard T, Nøjgaard C, Vind I, Krogfelt KA, Sørensen N (2019). Increased abundance of proteobacteria in aggressive crohn’s disease seven years after diagnosis. Sci Rep.

[22] Malham M, Lilje B, Houen G, Winther K, Andersen PS, Jakobsen C (2019). The microbiome reflects diagnosis and predicts disease severity in paediatric onset inflammatory bowel disease. Scand J Gastroenterol.

[23] Narula N, Kassam Z, Yuan Y, Colombel JF, Ponsioen C, Reinisch W, Moayyedi P (2017). Systematic review and meta-analysis: fecal microbiota transplantation for treatment of active ulcerative colitis. Inflamm Bowel Dis.

[24] Liu Y, Ajami NJ, El-Serag HB, Hair C, Graham DY, White DL, Chen L, Wang Z, Plew S, Kramer J, Cole R (2019). Dietary quality and the colonic mucosa–associated gut microbiome in humans. Am J Clin Nutr.

[25] Fernandez-Raudales D, Hoeflinger JL, Bringe NA, Cox SB, Dowd SE, Miller MJ, Gonzalez de Mejia E (2012). Consumption of different soymilk formulations differentially affects the gut microbiomes of overweight and obese men. Gut Microbes.

[26] Panasevich MR, Schuster CM, Phillips KE, Meers GM, Chintapalli SV, Wankhade UD, Shankar K, Butteiger DN, Krul ES, Thyfault JP, Rector RS (2017). Soy compared with milk protein in a Western diet changes fecal microbiota and decreases hepatic steatosis in obese OLETF rats. J Nutr Biochem.

[27] MacDermott RP (2007). Treatment of irritable bowel syndrome in outpatients with inflammatory bowel disease using a food and beverage intolerance, food and beverage avoidance diet. Inflamm Bowel Dis.

[28] Martin de Carpi J, Vila V, Varea V (2011). Application of the Porto criteria for the diagnosis of paediatric inflammatory bowel disease in a paediatric reference centre. An Pediatr (Barc).

[29] Lewis JD, Chuai S, Nessel L, Lichtenstein GR, Aberra FN, Ellenberg JH (2008). Use of the noninvasive components of the Mayo score to assess clinical response in ulcerative colitis. Inflamm Bowel Dis.

[30] Noordzij M, Tripepi G, Dekker FW, Zoccali C, Tanck MW, Jager KJ (2010). Sample size calculations: basic principles and common pitfalls. Nephrol Dial Transplant.

[31] Morshedzadeh N, Shahrokh S, Aghdaei HA, Pourhoseingholi MA, Chaleshi V, Hekmatdoost A, Karimi S, Zali MR, Mirmiran P (2019). Effects of flaxseed and flaxseed oil supplement on serum levels of inflammatory markers, metabolic parameters and severity of disease in patients with ulcerative colitis. Complement Ther Med.

[32] Forbes A, Escher J, Hébuterne X, Kłęk S, Krznaric Z, Schneider S, Shamir R, Stardelova K, Wierdsma N, Wiskin AE, Bischoff SC (2017). ESPEN guideline: clinical nutrition in inflammatory bowel disease. Clin Nutr.

[33] Szkaradkiewicz A, Marciniak R, Chudzicka-Strugala I, Wasilewska A, Drews M, Majewski P, Karpinski T, Zwozdziak B (2009). Proinflammatory cytokines and IL-10 in inflammatory bowel disease and colorectal cancer patients. Arch Immunol Ther Exp.

[34] Halmos EP, Christophersen CT, Bird AR, Shepherd SJ, Gibson PR, Muir JG (2015). Diets that differ in their FODMAP content alter the colonic luminal microenvironment. Gut.

[35] Flemer B, Lynch DB, Brown JM, Jeffery IB, Ryan FJ, Claesson MJ, O'riordain M, Shanahan F, O'toole PW (2017). Tumour-associated and non-tumour-associated microbiota in colorectal cancer. Gut.

[36] Merga Y, Campbell BJ, Rhodes JM (2014). Mucosal barrier, bacteria and inflammatory bowel disease: possibilities for therapy. Dig Dis.

[37] Vital M, Karch A, Pieper DH. Colonic butyrate-producing communities in humans: an overview using omics data. mSystems. 2017;2(6). 10.1128/mSystems.00130-17. 10.1128/mSystems.00130-17 PMC5715108 29238752

[38] Venegas DP, Marjorie K, Landskron G, González MJ, Quera R, Dijkstra G, Harmsen HJ, Faber KN, Hermoso MA (2019). Short chain fatty acids (SCFAs)-mediated gut epithelial and immune regulation and its relevance for inflammatory bowel diseases. Front Immunol.

[39] Wang W, Chen L, Zhou R, Wang X, Song L, Huang S, Wang G, Xia B (2014). Increased proportions of Bifidobacterium and the Lactobacillus group and loss of butyrate-producing bacteria in inflammatory bowel disease. J Clin Microbiol.

[40] Joossens M, Huys G, Cnockaert M, De Preter V, Verbeke K, Rutgeerts P, Vandamme P, Vermeire S (2011). Dysbiosis of the faecal microbiota in patients with Crohn’s disease and their unaffected relatives. Gut.

[41] Lee T, Clavel T, Smirnov K, Schmidt A, Lagkouvardos I, Walker A, Lucio M, Michalke B, Schmitt-Kopplin P, Fedorak R, Haller D (2017). Oral versus intravenous iron replacement therapy distinctly alters the gut microbiota and metabolome in patients with IBD. Gut.

[42] Dai C, Cao Q, Jiang M (2017). Clinical utility of fecal calprotectin monitoring in asymptomatic patients with inflammatory bowel disease. Inflamm Bowel Dis.

[43] Park KT, Heida A, van Rheenen PF (2017). Standardizing fecal calprotectin monitoring in asymptomatic patients with inflammatory bowel disease. Inflamm Bowel Dis.

[44] Kane SV, Sandborn WJ, Rufo PA, Zholudev A, Boone J, Lyerly D, Camilleri M, Hanauer SB (2003). Fecal lactoferrin is a sensitive and specific marker in identifying intestinal inflammation. Am J Gastroenterol.

[45] Verissimo R (2008). Quality of life in inflammatory bowel disease: psychometric evaluation of an IBDQ cross-culturally adapted version. J Gastrointestin Liver Dis.

[46] Peyrin-Biroulet L, Cieza A, Sandborn WJ, Coenen M, Chowers Y, Hibi T, Kostanjsek N, Stucki G, Colombel JF (2012). Development of the first disability index for inflammatory bowel disease based on the international classification of functioning, disability and health. Gut.

[47] Leong RW, Huang T, Ko Y, Jeon A, Chang J, Kohler F, Kariyawasam V (2014). Prospective validation study of the international classification of functioning, disability and health score in Crohn’s disease and ulcerative colitis. J Crohns Colitis.

[48] Neuendorf R, Harding A, Stello N, Hanes D, Wahbeh H (2016). Depression and anxiety in patients with inflammatory bowel disease: a systematic review. J Psychosom Res.

[49] Byrne G, Rosenfeld G, Leung Y, Qian H, Raudzus J, Nunez C, Bressler B (2017). Prevalence of anxiety and depression in patients with inflammatory bowel disease. Can J Gastroenterol Hepatol.

[50] Montazeri A, Vahdaninia M, Ebrahimi M, Jarvandi S (2003). The Hospital Anxiety and Depression Scale (HADS): translation and validation study of the Iranian version. Health Qual Life Outcomes.

[51] Schmitz N, Kruse J, Heckrath C, Alberti L, Tress W (1999). Diagnosing mental disorders in primary care: the General Health Questionnaire (GHQ) and the Symptom Check List (SCL-90-R) as screening instruments. Soc Psychiatry Psychiatr Epidemiol.

[52] Anjom-Shoae J, Sadeghi O, Hassanzadeh Keshteli A, Afshar H, Esmaillzadeh A, Adibi P (2018). The association between dietary intake of magnesium and psychiatric disorders among Iranian adults: a cross-sectional study. Br J Nutr.

[53] Sadeghi O, Hassanzadeh-Keshteli A, Afshar H, Esmaillzadeh A, Adibi P. The association of whole and refined grains consumption with psychological disorders among Iranian adults. Eur J Nutr. 2017. 10.1007/s00394-017-1585-x. 10.1007/s00394-017-1585-x 29189904

[54] Scheman A, Te R (2017). Contact allergy to salicylates and cross-reactions. Dermatitis.

[55] Zmudzinska M, Czarnecka-Operacz M, Silny W (2008). Contact allergy to glucocorticosteroids in patients with chronic venous leg ulcers, atopic dermatitis and contact allergy. Acta Dermatovenerol Croat.

[56] Wypych TP, Marsland BJ (2018). Antibiotics as instigators of microbial dysbiosis: implications for asthma and allergy. Trends Immunol.

[57] Nachvak SM, Moradi S, Anjom-shoae J, Rahmani J, Nasiri M, Maleki V, Sadeghi O (2019). Soy, soy isoflavones, and protein intake in relation to mortality from all causes, cancers, and cardiovascular diseases: a systematic review and dose-response meta-analysis of prospective cohort studies. J Acad Nutr Diet.

[58] Lee SM, Han HW, Yim SY (2015). Beneficial effects of soy milk and fiber on high cholesterol diet-induced alteration of gut microbiota and inflammatory gene expression in rats. Food Funct.

[59] Fernandes MA, Verstraete SG, Phan TG, Deng X, Stekol E, LaMere B, Lynch SV, Heyman MB, Delwart E. Enteric virome and bacterial microbiota in children with ulcerative colitis and Crohn’s disease. J Pediatr Gastroenterol Nutr. 2018. 10.1097/mpg.0000000000002140. 10.1097/MPG.0000000000002140 PMC6310095 30169455

[60] Luo S, Wen R, Wang Q, Zhao Z, Nong F, Fu Y, Huang S, Chen J, Zhou L, Luo X. Rhubarb peony decoction ameliorates ulcerative colitis in mice by regulating gut microbiota to restoring Th17/Treg balance. J Ethnopharmacol. 2018. 10.1016/j.jep.2018.08.033. 10.1016/j.jep.2018.08.033 30170079

[61] Ma X, Hu Y, Li X, Zheng X, Wang Y, Zhang J, Fu C, Geng F (2018). Periplaneta americana ameliorates dextran sulfate sodium-induced ulcerative colitis in rats by Keap1/Nrf-2 activation, intestinal barrier function, and gut microbiota regulation. Front Pharmacol.

[62] Brace C, Gloor GB, Ropeleski M, Allen-Vercoe E, Petrof EO (2014). Microbial composition analysis of Clostridium difficile infections in an ulcerative colitis patient treated with multiple fecal microbiota transplantations. J Crohns Colitis.

[63] Coscueta ER, Campos DA, Osório H, Nerli BB, Pintado M (2019). Enzymatic soy protein hydrolysis: a tool for biofunctional food ingredient production. Food Chem X.

[64] Radavelli BL, Zanella PB, Silva AS, Dall’Alba V (2018). Diet, microbiota and inflammatory bowel disease. Nutr Food Sci.

[65] Karczewski J, Troost FJ, Konings I, Dekker J, Kleerebezem M, Brummer RJ, Wells JM (2010). Regulation of human epithelial tight junction proteins by Lactobacillus plantarum in vivo and protective effects on the epithelial barrier. Am J Physiol Gastrointest Liver Physiol.

[66] Bai G, Ni K, Tsuruta T, Nishino N (2016). Dietary casein and soy protein isolate modulate the effects of raffinose and fructooligosaccharides on the composition and fermentation of gut microbiota in rats. J Food Sci.

[67] Butteiger DN, Hibberd AA, McGraw NJ, Napawan N, Hall-Porter JM, Krul ES (2016). Soy protein compared with milk protein in a western diet increases gut microbial diversity and reduces serum lipids in golden Syrian hamsters. J Nutr.

[68] Cabrera-Acosta GA, Milke-Garcia MP, Ramirez-Iglesias MT, Uscanga L (2012). Deficient lactose digestion and intolerance in a group of patients with chronic nonspecific ulcerative colitis: a controlled, double-blind, cross-over clinical trial. Rev Gastroenterol Mex.

[69] Ginard D, Riera J, Bonet L, Barranco L, Reyes J, Escarda A, Obrador A (2003). Lactose malabsorption in ulcerative colitis. A case-control study. Gastroenterol Hepatol.

[70] Svolos V, Hansen R, Nichols B, Quince C, Ijaz UZ, Papadopoulou RT, Edwards CA, Watson D, Alghamdi A, Brejnrod A, Ansalone C (2019). Treatment of active Crohn’s disease with an ordinary food-based diet that replicates exclusive enteral nutrition. Gastroenterology.

[71] Fiorino G, Sturniolo GC, Bossa F, Cassinotti A, Di Sabatino A, Giuffrida P, Danese S. A phase 2a, multicenter, randomized, double-blind, parallel-group, placebo-controlled trial of IBD98-M delayed-release capsules to induce remission in patients with active and mild to moderate ulcerative colitis. Cells. 2019;8(6). 10.3390/cells8060523. 10.3390/cells8060523 PMC6627752 31151306

[72] Sands BE, Joshi S, Haddad J, Freudenberg JM, Oommen DE, Hoffmann E, McCallum SW, Jacobson E (2016). Assessing colonic exposure, safety, and clinical activity of SRT2104, a novel oral SIRT1 activator, in patients with mild to moderate ulcerative colitis. Inflamm Bowel Dis.

[73] Dale HF, Jensen C, Hausken T, Hatlebakk JG, Brønstad I, Valeur J, Hoff DA, Lied GA. Effects of a cod protein hydrolysate supplement on symptoms, gut integrity markers and fecal fermentation in patients with irritable bowel syndrome. Nutrients. 2019;11(7). 10.3390/nu11071635. 10.3390/nu11071635 PMC6682970 31319590

