# Supplementary material for: Effects of soy milk consumption on gut microbiota, inflammatory markers, and disease severity in patients with ulcerative colitis: a study protocol for a randomized clinical trial
Source: Trials. 2020 Jun 23;21:565. doi: 10.1186/s13063-020-04523-8 (PMC7310397; doi:10.1186/s13063-020-04523-8)
Supplement: Supplementary file 1 — Additional file 1. [file 13063_2020_4523_MOESM1_ESM.docx]

**Supplementary file**

**A) Consent form**

**
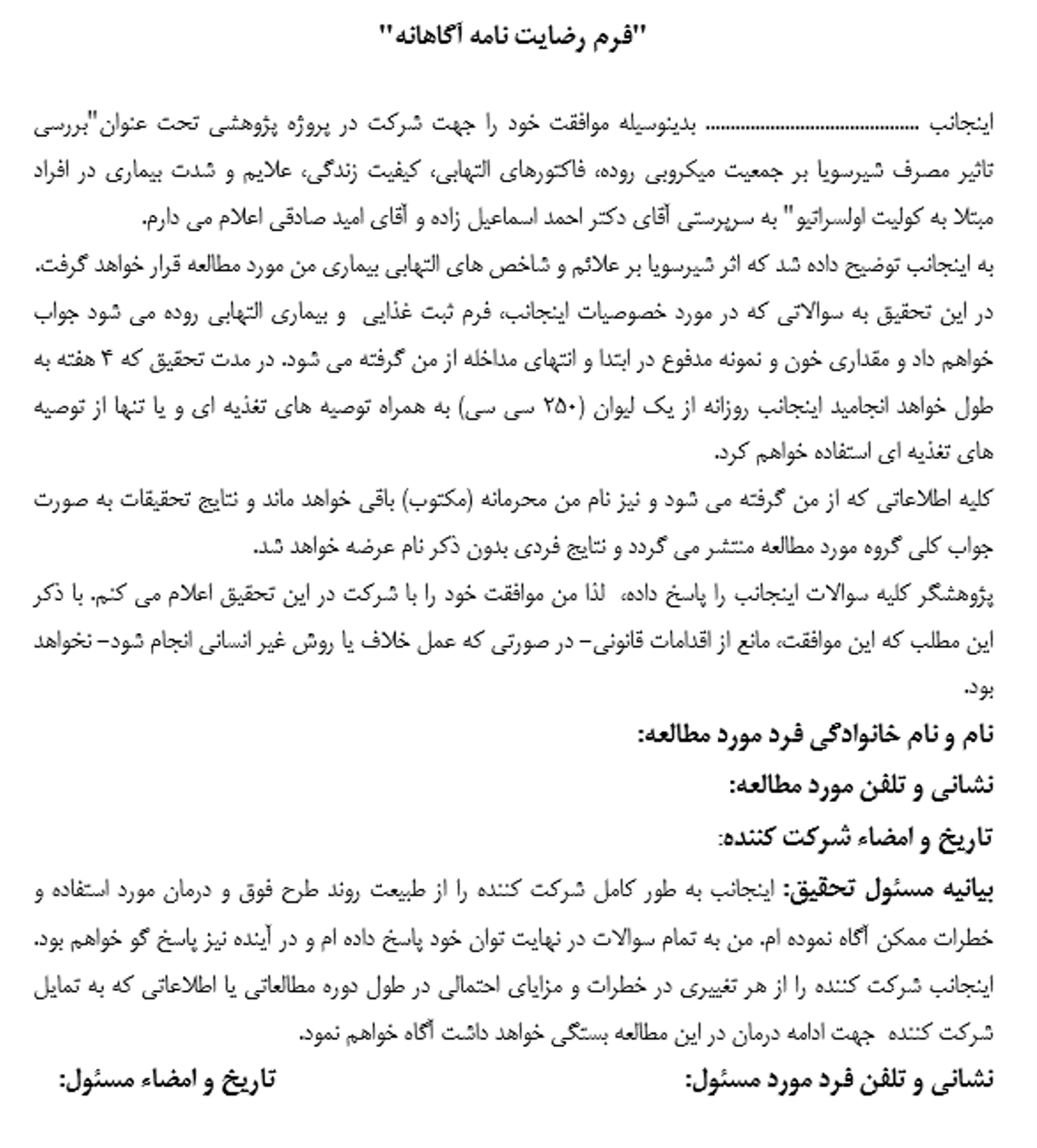
**

**B) Food recall**

| **Days** | **Meals** | **Time** | **Food items** | **Serving** | **Amount (gr)** |
| --- | --- | --- | --- | --- | --- |
| Day 1 (working day) |  |  |  |  |  |
|  |  |  |  |  |  |
|  |  |  |  |  |  |
|  |  |  |  |  |  |
|  |  |  |  |  |  |
|  |  |  |  |  |  |
|  |  |  |  |  |  |
|  |  |  |  |  |  |
| Day 2 (working day) |  |  |  |  |  |
|  |  |  |  |  |  |
|  |  |  |  |  |  |
|  |  |  |  |  |  |
|  |  |  |  |  |  |
|  |  |  |  |  |  |
|  |  |  |  |  |  |
|  |  |  |  |  |  |
| Day 3 (weekend) |  |  |  |  |  |
|  |  |  |  |  |  |
|  |  |  |  |  |  |
|  |  |  |  |  |  |
|  |  |  |  |  |  |
|  |  |  |  |  |  |
|  |  |  |  |  |  |
|  |  |  |  |  |  |

**C) Physical activity record**

| **Days** | **Activity description** | **Time** | **Duration** | **Intensity**  **(light, moderate, vigorous)** |
| --- | --- | --- | --- | --- |
| Day 1 (working day) |  |  |  |  |
|  |  |  |  |  |
|  |  |  |  |  |
|  |  |  |  |  |
|  |  |  |  |  |
|  |  |  |  |  |
|  |  |  |  |  |
|  |  |  |  |  |
| Day 2 (weekend) |  |  |  |  |
|  |  |  |  |  |
|  |  |  |  |  |
|  |  |  |  |  |
|  |  |  |  |  |
|  |  |  |  |  |
|  |  |  |  |  |
|  |  |  |  |  |

**D) 9-point partial Mayo score**

| Stool Frequency |  |
| --- | --- |
|  | 0 = Normal |
|  | 1 = 1–2 stools/day more than normal |
|  | 2 = 3–4 stools/day more than normal |
|  | 3 = >4 stools/day more than normal |
| Rectal bleeding* |  |
|  | 0 = None |
|  | 1 = Visible blood with stool less than half the time |
|  | 2 = Visible blood with stool half of the time or more |
|  | 3 = Passing blood alone |
| Physician rating of disease activity |  |
|  | 0 = Normal |
|  | 1 = Mild |
|  | 2 = Moderate |
|  | 3 = Severe |

^*^A score of 3 for bleeding required patients to have at least 50% of bowel motions accompanied by visible blood and at least one bowel motion with blood alone

The 9-point partial Mayo score was designed by Sutherland et al. (29)

**E) Inflammatory bowel disease questionnaire-9 (IBDQ-9) for quality-of-life assessment**

| Items | Scores | | | | | | |
| --- | --- | --- | --- | --- | --- | --- | --- |
|  | 1 | 2 | 3 | 4 | 5 | 6 | 7 |
| How frequent have your bowel movements been during the last 2 wk? | Bowel movements as or more frequent than they have ever been | Extremely frequent | Very frequent | Moderate increase in frequency of bowel movements | Some increase in frequency of bowel movements | Slight increase in frequency of bowel movements | Normal, no increase in frequency of bowel movements |
| How often has the feeling of fatigue or of being tired and worn out been a problem for you during the last 2 wk? | All of the time | Most of the time | A good bit of the time | Some of the time | A little bit of the time | Hardly any of the time | None of the time |
| How much energy have you had during the last 2 wk? | Most energy felt in years | Much more than usual | Slight increase | About the same | Slight decrease | Much less than usual | Wiped out |
| How often during the last 2 wk have you had to delay or cancel a social engagement because of your bowel problem? | All of the time | Most of the time | A good bit of the time | Some of the time | A little bit of the time | Hardly any of the time | None of the time |
| How often during the last 2 wk have you been troubled by cramps in your abdomen? | All of the time | A few times a day | Once a day | Every other day | Once/week | Twice/week | Never |
| How often during the last 2 wk have you felt generally unwell? | All of the time | Most of the time | A good bit of the time | Some of the time | A little bit of the time | Hardly any of the time | None of the time |
| Overall, in the last 2 wk, how much of a problem have you had with passing a large amount of gas? | A major problem | A big problem | A significant problem | Some trouble | A little trouble | Hardly any trouble | No trouble |
| How much of the time during the last 2 wk have you been troubled by feeling nauseated or sick to your stomach? | All of the time | Most of the time | A good bit of the time | Some of the time | A little bit of the time | Hardly any of the time | None of the time |
| How satisfied, happy, or pleased have you been with your personal life during the last 2 wk? | Very dissatisfied, unhappy mostly | Generally dissatisfied, unhappy | Somewhat dissatisfied, unhappy | Generally satisfied, pleased | Satisfied most of the time, happy | Very satisfied most of the time, happy | Extremely satisfied, could not have been more happy or pleased |

**F) Inflammatory bowel disease disability index (IBD-DI)**

**G) Hospital Anxiety and Depression Scale (HADS)**


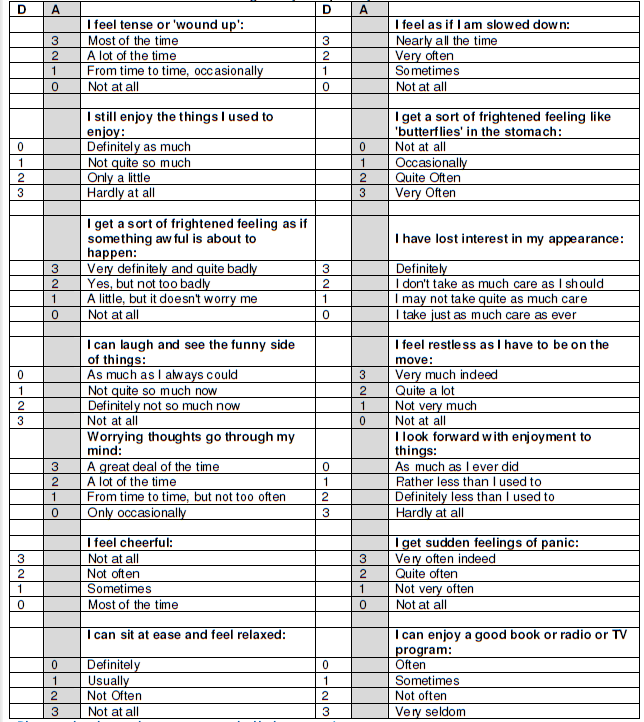


**H) General Health Questionnaire (GHQ 12)**

**Have you recently?**

| Questions | Answer options | | | |
| --- | --- | --- | --- | --- |
| Been able to concentrate on what you’re doing? | Better than usual | Same as usual | Less than usual | Much less than usual |
| Lost much sleep over worry? | Not at all | No more than usual | Rather more than usual | Much more than usual |
| Felt you were playing a useful part in things? | More so than usual | Same as usual | Less useful than usual | Much less useful |
| Felt capable of making decisions about things? | More so than usual | Same as usual | Less so than usual | Much less capable |
| Felt constantly under strain? | Not at all | No more than usual | Rather more than usual | Much more than usual |
| Felt you couldn’t overcome your difficulties? | Not at all | No more than usual | Rather more than usual | Much more than usual |
| Been able to enjoy your normal day-to-day activities? | More so than usual | Same as usual | Less so than usual | Much less than usual |
| Been able to face up to your problems? | More so than usual | Same as usual | Less so than usual | Much less able |
| Been feeling unhappy and depressed? | Not at all | No more than usual | Rather more than usual | Much more than usual |
| Been losing confidence in yourself? | Not at all | No more than usual | Rather more than usual | Much more than usual |
| Been thinking of yourself as a worthless person? | Not at all | No more than usual | Rather more than usual | Much more than usual |
| Been feeling reasonably happy, all things considered | More so than usual | About same as usual | Less so than usual | Much less than usual; |
